# Elder Family Financial Exploitation: The Complexity of Roles and Family Context

**DOI:** 10.1093/geroni/igaa057.1435

**Published:** 2020-12-16

**Authors:** Athena Chung Yin Chan, Marlene Stum

**Affiliations:** 1 University of Minnesota–Twin Cities, Saint Paul, Minnesota, United States; 2 University of Minnesota, Twin Cities, Saint Paul, Minnesota, United States

## Abstract

Despite elder family financial exploitation (EFFE) being recognized as the most prevalent type of elder abuse, little is known about the family context in which it occurs. To-date most EFFE research has focused on understanding the profiles of one victim and one perpetrator in reported cases. Informed by Family Systems Theory, this study offers new insight into the range and complexity of EFFE victim and perpetrator roles, family structures (relationship types across generations) and living arrangements. A mixed-methods dataset from a sample of non-perpetrator/non-victim concerned family members who experienced EFFE (most unreported to authorities) was utilized to map and analyze 23 family system genograms. The findings reflect four overall profiles when organized by the number of victim(s) and perpetrator(s) in each involved family system including: Single victim, single perpetrator (n=7), Single victim, multiple perpetrators (n=12), Two victims, single perpetrator (n=1), and Two victims, multiple perpetrators (n=3). Across the 4 profiles, most primary perpetrators moved in to live with the elder victims. For Single victim, single perpetrator cases, remarried spouses, as well as parent/adult child relationships in nuclear families with 2-3 adult children emerged. For Single victim, multiple perpetrators, up to five family perpetrators from 3 different nuclear families were involved, including adult children, their in-laws, and grandchildren as a common combination of perpetrators. The findings suggest EFFE is more complex than often assumed, involving multiple perpetrators and victims, and family relationship types beyond older parent/adult child. Implications for reframing risk profiles, assessment tools, and family-focused intervention strategies are discussed.

